# Assessing complementarities between live performances and YouTube video streaming

**DOI:** 10.1007/s00181-023-02444-4

**Published:** 2023-06-01

**Authors:** Juan D. Montoro-Pons, María Caballer-Tarazona, Manuel Cuadrado-García

**Affiliations:** 1 Department of Applied Economics, Universitat de València, València, Spain; 2 Department of Marketing, Universitat de València, València, Spain

**Keywords:** Spillover effects, Live music, Recorded music, Digitization, Regression discontinuity design, Gender gap, Z10, C14

## Abstract

Digitization and increased accessibility to recorded music have made revenue-generating activities increasingly tied to live performances. In this context, identifying the full impact of concerts (namely capturing the value of activities that emerge as a consequence of them) is of primary interest to assess the sustainability of the different music ecosystems. This paper analyzes spillover effects from playing live to YouTube video streaming. A sample of 190 artists performing in two international music festivals in years 2016 to 2019 has been selected, and the temporal patterns of online video searches for each one have been collected. Using a regression discontinuity design, results show a discrete jump of the YouTube search index for the average performer in the sample after playing live. Furthermore, there is evidence of a gender-specific effect: female performers experience a greater increase in YouTube searches. Though exploratory, this gender bias is consistent with potential theoretical explanations to be explored. Overall, findings provide causal evidence of the effect of live performances on a related but different market (i.e., recorded music), which underlines how technological disruptions may enable alternative revenue sources for musicians.

## Introduction

Digitization and the subsequent increased accessibility to content have challenged traditional revenue sources in the cultural sectors while new ones have emerged. In the case of music, this disruption has had a structural impact on the organization of the industry and the way in which audiences, musicians and intermediaries relate to each other (Hesmondhalgh and Meier 2018). Indeed, the last twenty years have witnessed a rapid transformation in music consumption and distribution where the decline of physical sales has been matched by an increasing relevance of live music attendance, and the streaming of digital contents has emerged as a business that has partially made up for the decline in recorded music sales (Montoro-Pons et al. 2021). Overall, technological innovation and change have transformed the landscape actors within the sector face.

The widespread use of new digital environments has facilitated interactions that open new and innovative ways of value creation and capture. Although the outcome of these may not be relevant in terms of their magnitude, they feedback and reinforce other revenue streams and, as such, should be considered as part of the wider music ecosystems (Hesmondhalgh 2021). The literature on the economics of the cultural industries has analyzed the impact emerging business models (especially in the music sectors) have on related products and/or services by creating complementarities or inducing substitutive effects. These can be broadly classified as within-market, mainly related to the effect on established business models as new ones are introduced, or cross-market, as spillovers across different (though related) products and/or services. The latter implies that cultural participation in one market can foster participation in a related, yet different, market. In this paper, we posit that live performances have a net positive effect on audiovisual (i.e., recorded) music consumption. These are spillover effects that emerge as a consequence of the exposure of consumers’ to new market information (through direct participation or indirectly by contagion) which increases the demand for a related market.

The focus of this research is to empirically examine cross-market effects from live music into audiovisual content viewing. While most of the literature focuses on the path from recorded music to live performances, we analyze the inverse link between both markets—i.e., the live-induced increased consumption of recorded music. In particular we analyze how playing live in a festival increases YouTube video searches, that we use as a proxy for the streaming of video content. YouTube, as the most used online music video content channel and a relevant source of information, plays a predominant promotional role for artists. In addition, with the fall of profits in the music industry, musicians draw on this channel as a revenue-generating source—from music-videos playbacks—as well as for developing new income streams (Edmond 2014).

This paper contributes to the understanding of cross-market effects in the creative industries, where interactions emerge between the physical and digital realms. It does so by reassessing the (more traditional or pre-filesharing) causal link between live performances and recorded music. Results show that playing live has a positive effect in a related market, that of streaming recorded content. To identify demand external effects on related markets with a clear causal direction is in itself non-trivial. To the best of our knowledge, there is no previous attempt to analyze the indirect effect of playing live to video streaming. Moreover, the variety of robustness checks we use provide strong support of the hypothesis of spillover effects from live to recorded music use.

The paper is organized as follows. We start by reviewing the literature on cross-market effects in the music sectors. Next, we discuss the empirical method and the dataset we use to test the main research hypothesis, i.e., the positive spillover effect from live performances on recorded music. Then, results are presented along with some robustness checks. Finally, we close with a detailed discussion of the results and some conclusions.

## Theoretical background

The impact between different business models in the music industry has been extensively assessed in the economics and management literature, with a focus on cross-demand effects emerging from the complementary or substitutive nature of marketed products and/or services. Contributions could be loosely classified according to whether they analyze within-market or cross-market effects. The former, i.e., effects are restricted to one market, deal mostly, albeit not exclusively, with the impact of new consumption ways of recorded music within the recorded music industry.

### Within-market effects in recorded music

Considering within-market effects in recorded music, Hendricks and Sorensen (2009) study the effect of product discovery in consumer demand. The authors analyze and find evidence supporting backward spillovers, that is, a positive effect of the release of a new album on an artist’s back-catalog. This effect stems from the enhanced diffusion of information between market participants associated with new albums, specially if these turn out to be a hit.

The emergence of legal online channels for digital music distribution has been found to have a negative effect on physical sales (Koh et al. 2014), as they reinforce the substitution of physical music by (legal) digital downloads and the trend toward unbundling—selling of individual tracks instead of full albums—(Elberse 2010; Papies and van Heerde 2017; Koh et al. 2019).

Streaming, as the main distribution channel of recorded music, has also attracted the interest of the academia. One early topic was that of the impact of streaming music services on other channels of recorded music distribution, i.e., the displacement of sales or cannibalization hypothesis. Nguyen et al. (2014) find that free streaming has no impact on CD sales. Wlömert and Papies (2016) use a panel of consumers to measure the impact of free (ads-based) and paid streaming services on music expenditures. They find that cannibalization does indeed occur, but its net effect is dependent on the type of service (whether it is free or fee-based) and consumer (active/inactive).

More recently, Aguiar and Waldfogel (2018) arrive to somehow conflicting results. From a market aggregate approach, they find that streaming displaces both permanent music downloads and music piracy. However, using a song-approach results are reversed: streaming increases sales and piracy. The latter, the authors claim, might be explained either to streaming actually increasing demand or to the presence of unobserved heterogeneity (which could invalidate the result).

Furthermore, the analysis of streaming platforms as new intermediaries in music (what has been labeled as the process of platformization of music, see Prey et al. 2022) stresses their curatorial power through playlists. These, in turn, contribute to the unbundling (and rebundling) of music (Bonini and Gandini 2019) with a potential deepening of substitution effects.

### Cross-market effects

When it comes to cross-market effects, authors have focused on the connection between live and recorded music markets. Krueger (2005) sets the agenda by stressing the decoupling between live and recorded music: the inflation in live tickets, the author claims, is a consequence of live music (in a post-filesharing world) being no longer a means of promoting recorded sales. In other words, a weakened complementary relation between live and recorded music weakens the magnitude of cross-market effects.

These findings open a new avenue of research in which the search for evidence goes in the opposite direction: from recorded to live music. In this new context recorded music would be an additional promotion means of live performances. Montoro-Pons and Cuadrado-García (2011) report evidence of an asymmetric cross-effect with the consumption of recorded music increasing the likelihood of live music attendance but not the other way around. In general, this line of research underscores the connection of media-based consumption with the increased demand for live performances. Mortimer et al. (2012) find a positive effect of file-sharing on live music (an effect that is more pronounced for smaller artists who see a relatively larger rise in awareness). Moreover, Nguyen et al. (2014) show that free music streaming affects positively live attendance.

Papies and van Heerde (2017) introduce a model to test diverse cross-format effects conditional on music/artists attributes and other market-related traits. While their model predicts a positive relation between concert revenue and record revenue and vice versa, these effects are mediated by different control variables. Overall they find a stronger effect of recorded music consumption on concert revenue than the inverse. Moreover their results turn out to be negative when certain mediators (such as piracy or unbundling) are considered, which suggests that the evidence supporting the connection between live and recorded music is, at best, ambiguous.

Christensen (2022) uses a natural experiment-like setting to evaluate the impact institutional disruptions have on live music demand. By drawing on the temporary removal in 2009 of Warner catalog on YouTube, the author finds a negative effect on live revenue for Warner acts. This suggests a complementarity in a performer’s ticket prices and streaming penetration.

So far, the literature reviewed underscores the impact of digital channels on live concerts. However, and as new business models settle, it may be reasonable to reassess the impact of live concerts on recorded music. By following this line, one acknowledges a transient decoupling between both markets, as new businesses and consumer practices develop. Here, the recent literature provides scant evidence.

Using aggregate data, Maasø (2018) analyzes the impact of a festival on music streaming. The authors leverage the data in *WiMP Music* (a subscription-based music streaming service that later became *Tidal*) to analyze a time series of streams before, during and after the 2010–2013 editions of the Øya festival in Norway. Findings support a 43% increase in streams of artists playing in the festival. However, the descriptive nature of the methodology falls short of determining whether this finding could be attributed to causality. Specifically, the fact that the streaming platform curates playlists associated with the festival lineup could cast doubts on the direction of the observed effect.

Drawing on individual-level data from the music website Last.fm Ternovski and Yasseri (2020) aim at identifying the direct and indirect impact of live attendance on streaming content. The authors extract those live events between 2013 and 2014 documented on Last.fm website and attendance as reported by users. Furthermore, they leverage data on friends of users who did not attend the live events to test the existence of peer or contagion (indirect) effects. A regression discontinuity design is applied to the time series of songs listened to by attenders three weeks before and after the event. Results show an increase of (roughly) 1 song per day per attendee which is consistent with the existence of a cross-market effect. As for the non-attenders, the effect is on average smaller, only found for the most popular artists and its magnitude depends on the number of Last.fm friends who actually attended.

Recently, Sim et al. (2022) analyze the negative impact of COVID-19 on music streaming. The authors conclude that restrictions in mobility implemented worldwide led to a substantial decrease in complementary activities (commuting or driving to cite two) to streaming music. While this finding is robustly validated, the hypothesis put forward in this paper—the causal link between live performances have on recorded music—could provide an additional explanation. As live performances were canceled due to lockdowns, a potential complementarity connection between live and streaming music was blocked.

Given that the balance of the music industry has shifted from the recorded music to live performances, it remains a challenge to identify ways in which the latter affects the former. This paper aims at further exploring this issue and testing the hypothesis of a positive impact of live performances on recorded music. To do so we benefit from an empirical approach that draws on the (online) user-generated data before and after a live event. A dataset composed of time series of a video search index across a sample of artists is used to test the existence of spillover effects from live to recorded music. Results agree with a positive impact of playing live with video search activity which is, on the other hand, consistent with an increase in video streaming. Our findings are robust and have a local and simple causal interpretation.

This paper aligns with that of Maasø (2018) in that aggregate data is used. Furthermore, and given the data source, estimates are consistent with joint evidence of direct and contagion effects—that is, searches performed by both attenders and non-attenders. However, the methodology is that used by Ternovski and Yasseri (2020) which, under specific conditions, allows to infer causal relationships from observational data. Nevertheless, we depart from them in that we consider a social media platform with a larger user base: YouTube ranks second (to Facebook) in number of users.^1^

## Methods and dataset

### Regression discontinuity designs, live performances and YouTube video searches

To quantify the (positive) spillover effects that live performances have on video streaming one must draw on causal inference methods. While randomized experiments provide the gold standard to measure the average effect of an intervention (playing live) on a response variable (video streaming), they are infeasible in many applications in the social sciences. If this is the case, researchers rely on observational data which, in turn, preclude the estimation of the kind of causal effect we are interested in unless some strict conditions are met or some additional information (such as the case of valid instruments) is provided (see Winship and Morgan 1999).

Regression discontinuity (RD) designs offer a quasi-experimental setup for making causal inferences using observational data. There is a growing literature using RD as one of the strongest non-randomized designs to estimate causal effects (see the survey by Villamizar-Villegas et al. 2021). Indeed, RD has been considered a valid tool to identify treatment effects with observational data sharing some properties of randomized trials (Angrist and Pischke 2009; Lee and Lemieux 2010; Imbens and Kalyanaraman 2012; Choi and Lee 2017).

We apply an RD design in the context of a sample of performers playing live to detect the influence, if any, on YouTube users’ behavior. In this case, the framework is that of an RD design in time, i.e., the running variable is time to an event, and we aim to estimate the causal impact of live performances on video streams. Note that we will not deal directly with video streams but use instead a YouTube searches index as a proxy.

The design works as follows: music video searches—$Y_{i,t}$, the outcome variable—are observed for performer *i* over a period of time. Having played live is the treatment status: at each time point performers have either played live ($D_{i,t}=1$) or not ($D_{i,t}=0$). The running variable $S_{i,t}$—time in weeks to the performance—determines whether unit *i* has undergone treatment, i.e., it splits units into treated and untreated at point *t*. Note the score $S_{i,t}$ is normalized to point to the intervention at the cutoff $S_{i,t}=t_i-c$, being $t_i$ time (week) at which outcome is observed and *c* the week at which performance occurs. Therefore it takes negative values ($S_{i,t}<0$) before a band plays live and positive ones ($S_{i,t}>0$) afterward. Finally, the value of the treatment $D_{i,t}$ is univocally affected by the running variable $S_{i,t}$, time to the event.

Define $\tau$, the average treatment effect, as the parameter of interest. Then, we propose the following specification

1 $$\begin{aligned} Y_{i,t}=a_i+D_{i,t}\, \tau + f(S_{i,t})+\epsilon _{i,t} \end{aligned}$$

being $f(\cdot )$ being a polynomial functional which is specified such that the regression function differs on both sides (*L* and *R*) of the cutoff:

$$\begin{aligned} f(S_{i,t})= & {} f_L(S_{i,t})+D_{i,t}(f_R(S_{i,t})-f_L(S_{i,t}))\\= & {} \beta _{L,1} S_{i,t}+\beta _{L,2} S_{i,t}^2+D_{i,t}S_{i,t} +\ldots \\ {}{} & {} +\,\, (\beta _{R,2}-\beta _{L,2}) D_{i,t}S_{i,t}+(\beta _{R,2}-\beta _{L,2}) D_{i,t}S_{i,t}^2+\ldots \end{aligned}$$

While the simplest way to implement RD is to fit the outcome on the score, we use artist-related covariates $X_{i,t}$ to improve efficiency. Then expression (1) turns into:

2 $$\begin{aligned} Y_{i,t}=a_i+D_{i,t}\, \tau + f(S_{i,t})+\gamma X_{i,t}+\epsilon _{i,t} \end{aligned}$$

Note that a local regression for expression (2) is proposed: only observations within a neighborhood (i.e., a bandwidth) of the cutoff are used to estimate $\tau$. Furthermore, a kernel is employed, so observations closer to the cutoff receive a greater weight.

All the analysis rests on the assumption that YouTube searches change abruptly due to performing live. Thus, the identifying assumption underlying the discontinuity approach is that in the absence of the event, YouTube searches related to bands playing would have changed smoothly. Empirical evidence will be consistent with the existence of spillover effect if the outcome is observed to discretely change at some threshold value of the score. Namely, is there a discrete jump in video searches once a band has performed live?

Furthermore, the appropriateness of an RD approach draws on the fact that its main assumptions hold. First, there is comparability of units around the cutoff. This might sound circular (Nick Cave is Nick Cave) but note that performers’ features evolve over time: released records, awareness, popularity and fan base... which may change how they are perceived by the public. Analyzing video searches on performers in a window around the cutoff (when the festival takes place), allows us to assume artists’ features remain unchanged and comparability applies. Second, there is no manipulation of the running variable. This is obvious as individuals do not affect dates of festivals which are set in advance well before the arrangement of the lineup takes place. Third, continuity of the relationship between the outcome and the running variable is assumed. It is reasonable to posit that once we remove the treatment (i.e., the live performance) there should not be a discontinuity in the relationship between outcome (YouTube searches) and running variable (time to performance).

Note that the local nature of estimates limits their external validity. However, this sits well with the analysis of the music market, where short life cycle and oversupply make the expected effects to be transitory. In other words increased video searches occur in a short window after the intervention takes place, i.e., searches are not permanently enhanced. However, this does not discard other permanent effects for the performer (e.g., performances could lead to an increase of the fan base).

Finally, RD designs where the score or running variable is time do have particularities that set them apart from the standard RD design. Note that, contrary to standard RD designs where the running variable splits subjects intro treated and non-treated, no randomization is at play as eventually all units (performers) become treated. Furthermore, sorting of the units around the cutoff has to be discarded. In our case one could consider the event occurs *as if* randomly as individuals cannot manipulate the date at which the event takes place—see Ternovski and Yasseri (2020) for a detailed discussion. The literature offers many examples of designs in which time is a running variable (Anderson 2014; Bahrs and Schumann 2020; Chenyihsu and Whalley 2012; Gallego et al. 2013). Besides, Hausman and Rapson (2018) offer a constructive critic and checklist on this type of designs. Additionally, we have resorted to enhanced robustness checks.

### The dataset

The dataset herein analyzed collects information on YouTube searches for performers in the 2016, 2017, 2018 and 2019 editions of two reputed music festivals in Spain: *Primavera Sound* and *Festival Internacional de Benic*à*ssim* (*FIB*). Choosing bands performing at two well established events simplified (and removed arbitrariness in) the decision on sampling units. Myriad of bands perform live in Spain every year and their heterogeneity is difficult to capture. By choosing bands performing at leading festivals, we assume they have achieved a certain reputation or have a reasonable expectation to become so. Furthermore, our empirical strategy draws on bands that generate enough Internet activity, something that is easier to achieve for bands associated with top ranking festivals. Indeed, both events were listed in the top ten music festivals in Spain using data available from the website of the Spanish association for music promoters.^2^ In addition, they offer a varied lineup proposal allowing us to capture the diversity of genres and styles of popular music and a reasonable mix of well-established and newer acts.

Prior to describing the dataset, we provide some details about the steps involved in the data collection process. First, we start by identifying the lineup of *Primavera Sound* and *FIB* in the selected years through web scraping. Ideally, for each band in the lineup a weekly index of YouTube searches is to be retrieved using *Google Trends*^3^ (GT). GT’s main functionality is that of providing a time series index of the volume of queries of any given search term in a specific geography. In our case, we used video searches in Spain. The index is produced from an unbiased sample of video search data over a week that is further translated into a normalized 0–100 scale. In this case the week with the maximum volume of queries is set as 100 and all other weeks are given relative weights. Data gathering was automated through the use of a *Python* application programming interface for GT.^4^

Second, the retrieval of information draws on the use of a search term which, in some instances, can be too vague or general. In order to disambiguate we resorted to GT’s *suggestions*, a functionality that allows to select queries that unambiguously refer to a specific search term (in our case a performer’s name). *Suggestions’* output is a (set of) key(s) or identifier(s). Each one links the different specific meanings related to a search term.^5^ In other words, each outcome comes with a set of identifiers that allow to explicitly refine the retrieved search. After inspection of the output only those identifiers that refer to artists/bands/performers are retained. Based on these, the sample was thus restricted to 246 bands for which a valid suggestion is generated.

Third, while theoretically the index can be retrieved at different frequencies (months, weeks, days and hours) in practical terms high-frequency data are only adequate for queries that generate a large amount of searches. On the contrary, search terms that do not generate enough searches in specific points in time are automatically assigned an index value of zero, which creates a downward-bias problem. To minimize this risk and, on the other hand, to be able to identify the time evolution of the index with a reasonable detail, we opt to retrieve the index for weekly data. Again, choosing a more precise time period, i.e., daily data, would mean many observations not generating enough searches, hence being assigned a value equal to zero. Once we retrieved the preliminary dataset we dropped those performers with more than 50% of zeroes for the index values in a window around the performance date of 8 weeks. This further reduced the sample to 190 performers.

Fourth, for each performer, roughly five years worth of search data are collected: each time series spans a period that starts 253 weeks before the festival takes place and ends 6 weeks after it. However note that the local nature of the RD design estimates implies that only those observations around the cutoff date (i.e., the live performance) will be used. Nevertheless, we make use of observations further apart from the cutoff for robustness checks.

Finally, the RD design’s estimation procedure will make use of additional (band-level) covariates to enhance the efficiency of the estimates (Calonico et al. 2019). Specifically, the number of years since first album was released (and its square) and genre of the band (a dummy which takes on value 1 if the band is classified as *pop* or *rock* in the website https://www.pitchfork.com) are included. Moreover, a trend line is added to control for time patterns. Table 1 summarizes some of the features of the sample. It includes number of bands, average year of first release and most frequent genre-distribution. Summaries are provided for the full sample as well as for the individual festivals.

**Table 1:** Summary of the sample

	Primavera sound	FIB	Full sample
Number of performers	107	83	190
First album (average year)	2004	2007	2005
*Genre (% of performers)*			
Rock	52	59	55
Pop	31	22	27
Electronic	16	28	21
Rap, Hip-hop	15	13	14
RnB	17	10	14
$\hbox {Gender}^*$	36	15	27

$^*$Percent of female performers or bands fronted by women

## Results

### Selecting the running variable

Before introducing the results, we need to clarify some design choices regarding the running variable. The structure of the dataset is a panel following bands/performers over time. It contains each performer’s YouTube weekly search index (the outcome), a set of covariates, and a transformation of the time variable (the running variable). Note that events (performances) take place in a specific point in time (a day) that differs from the scale of the time-variable we use (weekly aggregate data on searches). This implies some design decisions with regard to the running variable.

Performances take place within events, i.e., festivals. For each festival edition, the start and end dates are known. In this regard, we need to make some clarifications regarding the specific time stamp of the video search index. First, the weekly search index collects information of the week where the starting point is always Sunday. For instance, the index at date 2019-09-01 refers to YouTube searches in the week starting Sunday September 1 and ending Saturday September 7 (year 2019). This means the index summarizes forward-looking searches at the specific date it points.

Second, for all performers we collect the start and end dates of the festival edition they play at. These dates are then used to build the running variable. However, the use of weekly data introduces an ambiguity to the choice of cutoff date. Choosing the end of the festival as cutoff (always on Sunday) means the index at the cutoff retrieves video searches on the week following the performance. Alternatively, choosing the start of the festival (on Wednesday or Thursday) the cutoff would point to somewhere in between the actual start and the week after the festival. To actually retrieve information on the week a performance takes place we need to move the pointer to the Sunday before the Wednesday/Thursday the festival starts. By shifting it slightly backward, treated observations are always located to the right of the cutoff. In practice we use all the three cutoffs to check the robustness of the estimates.

**Fig. 1:**
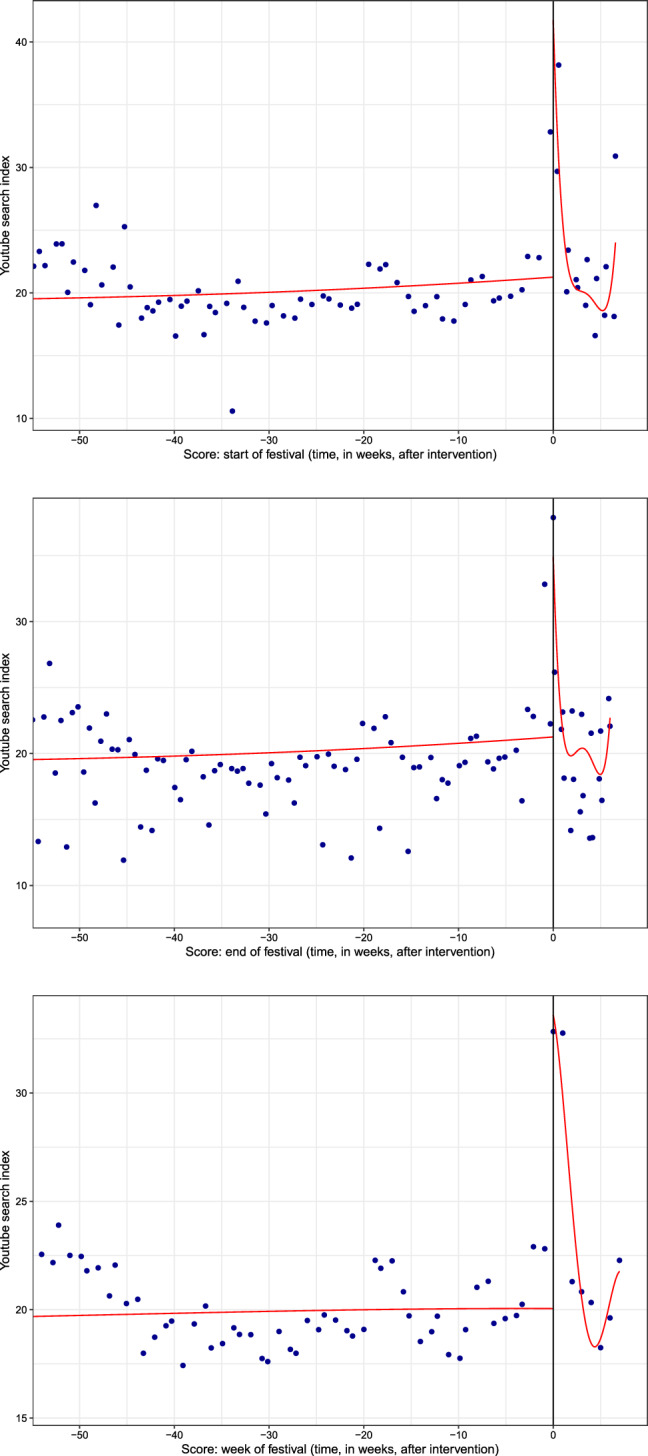
Regression discontinuity plots for different adjustments of the running variable. Cutoff point is: start of the festival (top); end of the festival (middle); week of the festival (bottom)

As a graphical aid to identify the existence of an effect of playing live on video searches, Fig. 1 shows the discontinuity plots using the three running variables. Data are binned across the running variable and means for the observations within each bin are computed (dots in the plots). In this case, the number of bins was data-driven,^6^ and the resulting smoothed local regression line is shown. A discrete jump in the cutoff date becomes apparent in the three plots, which becomes more evident when using week of the festival as the score. The next subsection provides estimates for this observed effect.

### Estimating the effect

The *R* package *rdrobust* (Calonico et al. 2021) has been used to estimate the effect through a nonparametric local polynomial approach. In this respect, there are two choices to make: the kernel (weights) used in the estimation and the bandwidth that controls the neighborhood around the cutoff that is to be used in the estimation. As for the weights, a triangular kernel has been employed, although results are robust to alternative kernels. With regard to the bandwidth, we resort to a data-driven approach (Calonico et al. 2016) by choosing a bandwidth that minimizes the mean square error (MSE) of the RD point estimator. We allow bandwidth to differ on both sides of the cutoff given the asymmetric length of the time series. However, results remain qualitatively unchanged when the same bandwidth is used on both sides. In addition, results were robust to changes in the pre-event window of the time series.^7^

Furthermore, we control for non-independent residuals due to the panel structure of the data by clustering the observations by performer. In addition, the efficiency of the estimator can be increased by introducing covariates. Specifically we control for: (i) time (in years) since a band/performer released its first recording (and its square); (iii) genre of the performer through a binary variable to indicate whether a performer is classified as pop/rock in pitchfork.com; and (iii) a trend to account for the dynamics of the data. Note none of these covariates are expected to differ at the cutoff; hence, they are balanced and further (restrictive) assumptions are not needed.

**Table 2:** Estimate results: increase in YouTube search index after treatment

Method	Coeff	SE	*z*-val	P$>|z|$	CI lower	CI upper
*1. Cutoff: start of festival*						
Conventional	8.77	1.99	4.41	0.00	4.87	12.67
Robust		2.52	5.47	0.00	8.85	18.74
*2. Cutoff: end of festival*						
Conventional	8.12	1.89	4.30	0.00	4.42	11.82
Robust		2.20	5.13	0.00	6.98	15.61
*3. Cutoff: week of festival*						
Conventional	10.73	2.15	4.98	0.00	6.51	14.95
Robust		3.23	6.26	0.00	13.90	26.57

Table 2 summarizes the main results. In addition to conventional 95% confidence interval estimators—based on the MSE-optimal point estimator—it provides bias-corrected confidence intervals.^8^ Note that inference based on the conventional (and optimal) point estimator is not desirable as it assumes the local polynomial provides an exact approximation to the true underlying regression function. Robust (bias-corrected) estimates lead to improved coverage in finite samples and result in valid inferences when optimal MSE-bandwidth is used.

**Fig. 2:**
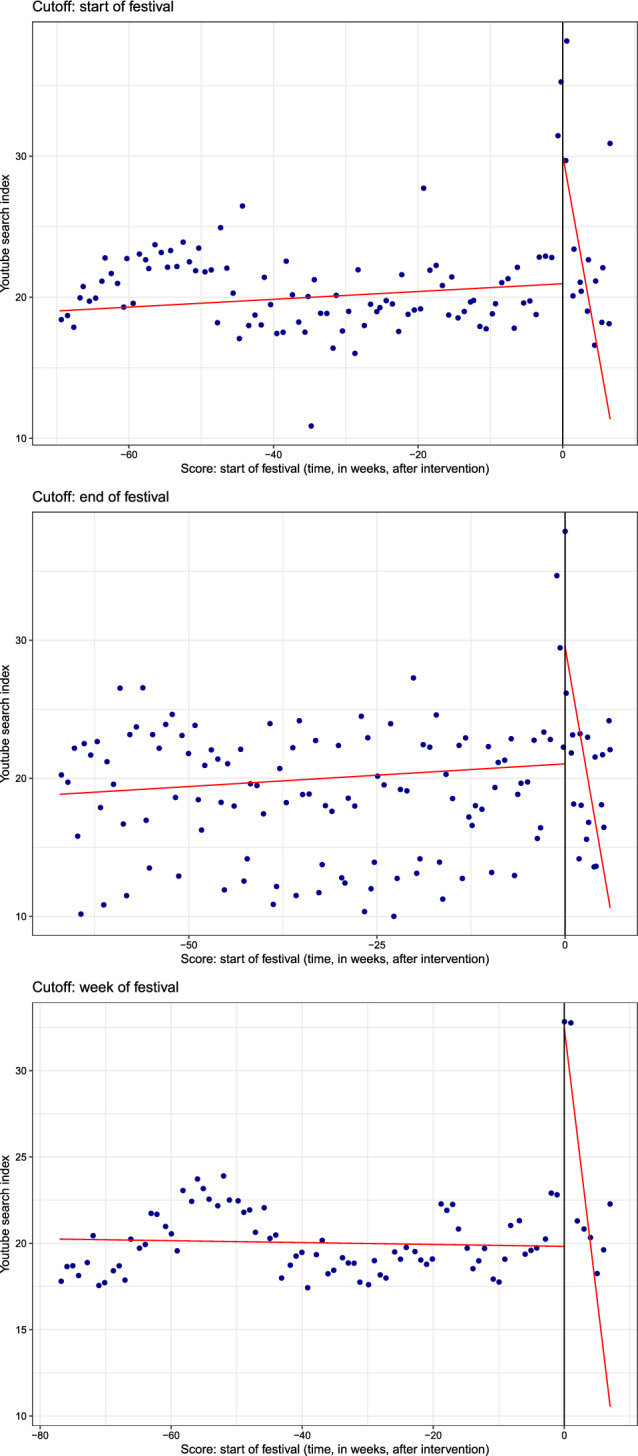
Estimates of the discontinuity effects using a local polynomial regression

Overall, results provide evidence consistent with the effect of playing live on the index of video searches, which experiences an increase ranging from 8.12 to 10.73 points. While the magnitude of the effect is affected by the choice of the cutoff, robust bias-corrected intervals suggest a positive treatment effect in all cases. Note these results are also found when pooled data are used (without clustering of observations) and no covariates are included in the model. Graphically, Fig. 2 plots the RD estimates in the three different scenarios and provides a visual account of the positive jump (i.e., the discontinuity) in video searches at the time performances take place. This, in turn, delivers visual evidence of spillover effects emerging from live performances to the videostreaming of recorded music.

### Conditional effects: genre and gender of performer

Next, we analyze the treatment effect conditioning on specific covariates of interest. In particular we estimate the effect across: (i) genre of performer and (ii) gender of the performer. The goal of this exercise is to identify (if any) potential differences in audiences response to performers when looking at two classification criteria.

Genres can be seen as institutional devices that organize supply and demand in cultural markets. They help position artists and, in so doing, provide signals to potential audiences and help set expectations to market participants (Lena and Peterson 2008). Interestingly, genres have been found predictors of commercial success (Askin and Mauskapf 2017), which makes them a good candidate to be used as a source of differential spillover effects. Without imposing any a priori assumption on how genre mediates spillovers, and given the restricted variability in music genres in the sample we use a binary classification were we subset the sample into performers classified as either Rock or Pop and the remainder. Roughly 66% of the performers fall into the pop/rock class.

The approach to gender is more contentious, and results are to be considered of a more exploratory nature. Again, we expect it to influence supply and demand in the music sectors. Considering demand, the literature on cultural consumption has found relative support for different headline participation rates across genders. Furthermore differences have been found when accounting for high-brow/low-brow participation. For instance, women have been found to have lower participation rates for both live and prerecorded music (Montoro-Pons and Cuadrado-García 2011) and to show a tendency for being omnivorous—i.e., showing a higher propensity to participate in both high-brow and low-brow manifestations (Favaro and Frateschi 2007).

On the other hand, and to the best of our knowledge, no study on the role of gender on spillovers in cultural supply is to be found in the literature. Nevertheless, the scant presence of female performers in the music industry has been brought to the center of the debate in recent years. A study in the UK found that 70% of performers of lineups of festivals in 2017 were male or all-male bands (Sherlock and Bradshaw 2017). In a similar vein Mitchum and García-Olano (2018) find that in 2018 70% of the artists across the largest US festivals were male (down from 75% in 2017). As a response, an initiative from within the industry aiming at achieving gender-balance is gaining momentum with a few relevant players (such as *Primavera Sound* starting in its 2019 edition) already sticking to a 50:50 gender split.^9^ Do these gender differences translate into asymmetric effects? To analyze the role of gender we condition the sample on the gender of the performer or (if a band) the genre of the individual who fronts the band.

Results for both exercises are shown in Tables 3 and 4. With regard to genre, estimated effects are significant and found to be greater for pop/rock performers who experience a 14 points increase in the search index compared to 8.61 by *other genres*. This finding is consistent with the hypothesis of a differential impact of genres, with pop/rock having a broader appeal to the general public. In addition, female performers (or bands fronted by females) generate more video streaming activity (increase in 14.6 points) than non-female ones (11.3 points). Both results suggest some interesting lines of work. Findings indicate a clear match between pop/rock and female performers and YouTube video streaming. In the case of genre, we find support to the hypothesis of the broader appeal of pop to audiences. As for female acts, the fact they could generate more online activity than male performers opens an interesting research avenue worth of exploring.

**Table 3:** Differential effects across genre

Method	Coeff	SE	*z*-val	$P>|z|$	CI lower	CI upper
*Subsample: pop/rock performers*						
Conventional	14.03	2.48	5.66	0.00	9.17	18.89
Robust		2.76	5.95	0.00	11.02	21.84
*Subsample: other genres*						
Conventional	8.61	3.22	2.68	0.01	2.31	14.91
Robust		3.30	2.93	0.00	3.22	16.17

**Table 4:** Differential effects across gender of performer

Method	Coeff	SE	*z*-val	$P>|z|$	CI lower	CI upper
*Subsample: female performers*						
Conventional	14.60	3.95	3.69	0.00	6.85	22.35
Robust		4.31	3.91	0.00	8.41	25.29
*Subsample: male performers*						
Conventional	11.30	2.24	5.06	0.00	6.92	15.68
Robust		2.45	5.38	0.00	8.38	17.97

### Conditional effects: popularity of the performer

One could question whether the observed effects are asymmetric across different levels of popularity of performers. Theoretically, the expected conditional effect is ambiguous. As noted in the literature review, evidence has been found of spillovers from file-sharing to live demand being stronger for less successful artists, as they could benefit most from the raised awareness (Mortimer et al. 2012). Alternatively, one might argue that most popular performers should create more buzz and therefore generate more online activity generated after a performance.

To provide evidence on this research question, we recover the effect of live performances on video searches conditional on the popularity of the performer. In this regard, we must first define a measure of success or popularity. Note that success could be measured on different dimensions. We have come up with a feasible and simple alternative: using the number of results a Google search for a performer returns.^10^ The rationale is simple: the more popular an artist is, the more search results is should produce.^11^

Using the distribution of search results one can classify the sample across different quantiles. In particular we used three different classifications: (1) top 25% performers vs. bottom 75%; (2) top 50% vs. bottom 50%; and (3) top 33% vs. bottom 66%.

**Table 5:** Differential effects across the distribution of performer’s popularity

Method	Coeff	SE	*z*	$P>|z|$	CI lower	CI upper
*Subsample: top 25%*						
Conventional	12.60	3.26	3.87	0.00	6.22	18.99
Robust		3.45	4.21	0.00	7.76	21.30
*Subsample: bottom 75%*						
Conventional	11.98	2.33	5.15	0.00	7.42	16.54
Robust		2.61	5.40	0.00	8.98	19.21
*Subsample: top 50%*						
Conventional	12.13	2.33	5.20	0.00	7.56	16.70
Robust		2.57	5.51	0.00	9.13	19.21
*Subsample: bottom 50%*						
Conventional	12.52	3.19	3.92	0.00	6.27	18.78
Robust		3.51	4.00	0.00	7.18	20.95
*Subsample: top 33%*						
Conventional	12.49	2.90	4.30	0.00	6.80	18.18
Robust		3.19	4.63	0.00	8.52	21.04
*Subsample: bottom 66%*						
Conventional	11.89	2.45	4.86	0.00	7.09	16.69
Robust		2.70	5.11	0.00	8.51	19.10

Table 5 shows the findings from this exercise. Results do not provide strong evidence of asymmetric effects. Indeed neither sample split has produced remarkable differences regarding the magnitude of the estimated spillovers. While marginally larger point estimates were found for more popular acts in the 25–75 and 33–66 splits, these results were reversed when partitioning the sample using the median. Anyhow, as it can be seen, differences in magnitude are rather thin.

## Validation of the results

The literature on RD designs provides alternative strategies to validate empirical results (Thoemmes et al. 2017). In our case, different approaches have been undertaken.

First, a placebo cutoff strategy has been followed. Different artificial cutoffs have been selected from roughly one year (52 weeks) to one month (4 weeks) before the actual performance takes place. Note that choosing arbitrary cutoffs should produce non-significant estimates and therefore support the estimated effects in Table 2. Conversely, if estimated effects for this artificial interventions were significant, they would challenge the reliability of the results.

As for the rationale of the specific artificial cutoffs chosen, note that end of spring-beginning of summer marks the start of the tour season for many performers and that evidence of a seasonal pattern in live music consumption has been consistently found in the literature (Montoro-Pons and Cuadrado-García 2016). Could this drive a general interest in music regardless of the specific performers we choose? If so, one could find a peak in the public interest/awareness in music to be coupled with music video searches happening around the start of summer—roughly 52 weeks before the live performances considered—regardless of whether actual live performances are taking place. As for the other placebo cutoffs—26, 12 and 4 weeks before the event—they might be capturing announcement effects, promotion efforts by the festivals and/or anticipation to the actual event by consumers.

**Table 6:** Validation (i): estimation for alternative placebo cutoffs

Method	Coeff	SE	*z*-val	$P>|z|$	CI lower	CI upper
*Cutoff: week *=−*52*						
Conventional	$-$0.46	1.81	$-$0.25	0.80	$-$4.00	3.08
Robust		1.95	$-$0.27	0.79	$-$4.35	3.29
*Cutoff: week *= −*26*						
Conventional	1.72	1.60	1.08	0.28	$-$1.41	4.86
Robust		1.65	1.95	0.05	$-$0.02	6.46
*Cutoff: week *=−*12*						
Conventional	$-$0.77	1.65	$-$0.47	0.64	$-$4.01	2.47
Robust		1.75	$-$0.49	0.62	$-$4.29	2.57
*Cutoff: week *=−*4*						
Conventional	3.07	1.68	1.82	0.07	$-$0.23	6.38
Robust		1.78	$-$0.34	0.73	$-$4.10	2.89

**Fig. 3:**
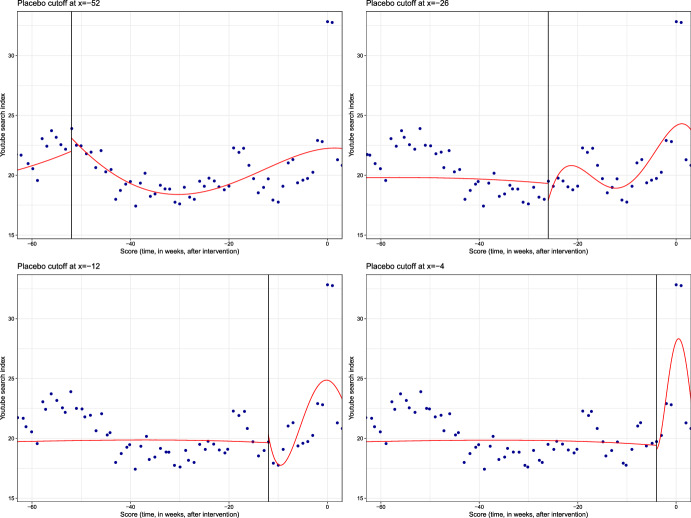
Regression discontinuity plots with artificial cutoffs at roughly) one year (top left), six months (top right), three months (bottom left) and one month (bottom right) before actual cutoff

Table 6 shows the results for different placebo cutoffs when using week of the festival as running variable, while Fig. 3 displays the RD plots. Setting the cutoff one year to one month before the actual intervention takes place we cannot conclude with significant effects using the standard 5% significance level. In all cases, the effect is indistinguishable from zero as the estimated confidence intervals (both conventional and robust) cross the zero line. Furthermore, the small magnitude of the estimated effects adds to the evidence supporting the findings in the previous section.

An additional placebo test was run. In this case, we want to rule out alternative explanations why a performer should see an increase in YouTube searches. Here, we could find performer-specific characteristics, such as new album or video releases, or other external factors such as seasonal confounding that makes audiences more prone to music in general.

To do so we have collected data on the same sample of performers for the same time periods but changing the geography from which searches were run: in this exercise we use YouTube searches in the US. If the estimated effects are causal, then results of this exercise should be non-significant. Table 7 shows the resulting effects for the alternative cutoffs. In it, no effect is significantly different from zero which reassures the existence of a causal effect from playing live.

Second, we have analyzed the sensitivity of the solutions to bandwidth choice. Results are shown in table 8 using week of the festival as the running variable. Besides the MSE-optimal bandwidth we have estimated a coverage error (CER) optimal bandwidth (Calonico et al. 2019). For each one, symmetric—equal width to the left and right of the cutoff—and non-symmetric bandwidths have been used. Furthermore, twice the optimal bandwidths were used. As it can be seen, all results are qualitatively similar and produce estimated effects that are robust to bandwidth specification.

Finally, to validate the results we run some tests to check the density of the running variable and used predetermined covariates as outcomes, i.e., placebo outcomes. All supported the robustness of the findings and are not included for the sake of brevity and given these tests (used in the literature) are less meaningful in this specific case. The nature of the running variable (time) implies no possibility of sorting around the cutoff; hence, its density should be balanced around the threshold. As for the continuous covariates (years since first release and a trend variable), unsurprisingly they did not show a discontinuity at cutoff.

**Table 7:** Placebo test using an alternative geography

Method	Coeff	SE	*z*	$P >|z|$	CI lower	CI upper
*1. Cutoff: start of festival*						
Conventional	0.22	1.17	0.18	0.85	$-$2.08	2.51
Robust		1.76	0.28	0.78	$-$2.95	3.94
*2. Cutoff: end of festival*						
Conventional	0.69	1.08	0.64	0.52	$-$1.43	2.81
Robust		1.40	0.86	0.39	$-$1.54	3.94
*3. Cutoff: week of festival*						
Conventional	0.52	0.99	0.52	0.60	$-$1.43	2.46
Robust		1.23	0.45	0.66	$-$1.86	2.96

**Table 8:** Validation (ii): sensitivity to bandwidth choices

Method	Coeff	SE	*z*-val	$P>|z|$	CI lower	CI upper
*One common MSE-optimal bandwidth*						
Conventional	8.90	1.88	4.74	0.00	5.22	12.58
Robust		2.15	5.99	0.00	8.68	17.13
*One common CER-optimal bandwidth*						
Conventional	8.85	1.88	4.71	0.00	5.17	12.53
Robust		2.15	5.96	0.00	8.60	17.03
*Two different MSE-optimal bandwidth (left/right of cutoff)*						
Conventional	12.89	2.04	6.31	0.00	8.89	16.90
Robust		2.15	6.46	0.00	9.66	18.08
*Two different CER-optimal bandwidth (left/right of cutoff)*						
Conventional	12.25	1.97	6.22	0.00	8.39	16.11
Robust	14.22	2.15	6.63	0.00	10.01	18.43
$2\times$!*MSE-optimal bandwidth*						
Conventional	8.96	1.87	4.78	0.00	5.29	12.63
Robust	12.80	2.15	5.96	0.00	8.59	17.02
$2\times$ *CER-optimal bandwidth*						
Conventional	9.90	1.86	5.32	0.00	6.25	13.55
Robust		2.15	5.96	0.00	8.62	17.05

## Discussion of the results

Business model innovations have deeply changed value creation in the music industry, altering traditional revenue streams and facilitating new ones. Such is the case of offline/online cross-market effects. This paper has tested the hypothesis of spillover effects going from live music to prerecorded music (video streaming through an online platform). To do so, an index of YouTube video searches is analyzed for a sample of performers at different editions of two established music festivals in Spain.

Results support the cross-market effect hypothesis: after playing live the index of video searches increases by roughly 9–10 points with the robust confidence intervals being in most cases well above this amount (see Table 2). Findings are, by and large, consistent with the hypothesis of positive spillover effects from live to recorded music. This, we believe, is a non-trivial contribution in itself as the evidence so far about this type of effects is scant and/or inconclusive. We add to recent evidence identifying the causality link from live to recorded music—which sets this work apart from Maasø (2018). Besides, we use recent data on video streams from one of the largest social media platforms and analyze performer traits that could condition video streams, namely genre and gender.

Overall, findings support the connection between the two markets (live and recorded), a link that can be transformed into a revenue source for performers. This stems from the fact that findings refer to YouTube videos, which artists do monetize directly—as they are, at least partially, right holders—but also potentially in an indirect way—through an increased awareness that can help raise artists’ profiles, expand their fan base and increase other revenue-generating consumer-related activities such as sound streaming.

From a methodological standpoint, results were reached through an RD design, which can be seen as a quasi-experimental method that, provided some conditions are met, produces valid causal inferences. In this research, findings provide evidence of a causal effect from live to prerecorded music. The fact that, after plotting the dataset, a discontinuity in the outcome emerges around the time the festivals take place (i.e., artists perform live) is suggestive of the adequacy of the RD design and provides a powerful graphical tool to illustrate the results. However, given in an RD design in time all units eventually become treated, a thorough set of robustness tests have been run which further have supported the results.

### The economic value of (increased) YouTube searches

One could question whether the economic value brought about by increased YouTube searches is of relevance. In this regard, we must note that the outcome in this design is an index (not the number) of YouTube searches. It measures searches relative to its maximum value (where it achieves a value of 100) over a period of time. Therefore, for the sample of performers, we are using a relative measure of searches and not their actual number (something that GT does not disclose). Having said so, an increase in the search index means a jump in actual searches (whatever this value may be). Then, assuming most searches are goal oriented, that is, one searches a video to play it back, the number of actual video streams must also jump. The question at stake is that of the economic value of searches.

First, one should note there is a direct revenue stream that emerges from video playbacks through ad and subscription revenue sharing. This is what we could identify as the short-term effect. In this respect, Soha and McDowell (2016) state that “YouTube splits ad revenue with the rights holder [...] somewhere between 40% and 50%”. Unfortunately, it is not simple to come up with a figure for the cost per 1000 views a YouTube video generates: it depends among other things on country of origin of the view and type of video. Anyhow as with any other revenue source from recorded music, that amount is split between music label, songwriters and publishers.

Furthermore, one must note that we are dealing with searches (and not actual playbacks) that can potentially generate multiple streams over time. For instance if a user after running a search includes a video in a playlist or replays it through their playback history. In this case the actual effect could last longer (and its economic value be greater) than the short-term impact we measured through search activity. In other words, while we have found a transient effect on search activity after a performance, the streaming effect could extend for a longer time period.

Second, besides the economic value associated with video streams, one should consider additional longer-term effects. We have already mentioned an increased fan base, an effect that builds up from live performances and which is not restricted to actual audiences but expands beyond these (through a contagion effect) through the media and social networks enabling discovery and increased awareness making bands better known to the public and reducing barriers they face to reach audiences. Here, we must point out the unconstrained nature of the data (geography includes all YouTube searches in Spain), implying we are looking at the amplified effect that a performance has on potential audience including attendees (the actual audience) who are positively affected through a direct exposure to the event and non-attendees (or indirect audiences) who are affected through exposure through traditional media (TV, newspapers and the so) plus social networks and eWOM. As Lee and Kim (2022) note, success in recorded music involves a mix of an artist’s talent, the influence of record labels or platforms and the influence of all stakeholders, especially consumers. This refers to the process of content production, distribution and consumption, where consumers influence through user-generated content such as e-WOM.

Additionally, one must note that for those performers signed to a label, a boost in YouTube playback activity is linked to increasing copyright claims (for performers but, especially, for record labels). This, in turn, raises the market value of the creators (band/performer) as an asset in the portfolio of bands of the labels that sign them, which, in turn, could sustain or boost a career with the record label.

All in all, it is reasonable to assume increased searches set in motion both short-term effects—video streams that go beyond the time frame in which the increase in searches is observed—and longer-term effects—namely audience enlargement through discovery, increased awareness and eWOM, or increased band value to record label and/or platforms.

### The gender effect

Furthermore, results conditional on genre and gender of the performer were obtained. As for genre, results are in line with estimations by Askin and Mauskapf (2017), who find that pop songs reached higher position and stayed longer on charts. Translated into our framework, this broader appeal of pop carries a greater spillover effect.

More interestingly the gender effect opens a potentially fruitful line of research. Indeed, our findings are consistent with a gender bias gap, worthy of further research. However, given the nature of the data set at hand, these results are to be considered of an exploratory nature and as such rather preliminary.

Three alternative explanations are consistent with the observed evidence. On the one hand, it could be a case of discovery and visibility at play where live events help increase the public awareness of women musicians more than that of their male counterparts. The fact that women are systematically underrepresented in the typical festival lineup—a situation which is not much different from those found in other labor settings (Lutter 2015)—could make audiences learning about female acts more impactful. Lack of visibility and recognition of women in music constrains female musicians’ opportunities to showcase their talent and reach wider audiences. Once the right conditions are set, e.g., playing to audiences of reputed events, awareness is triggered. In addition, institutional attempts at leveling the field could also add to the impact by bringing women performers to the front of lineups.

Anecdotal evidence suggests discovery to be associated with female musicians in other music industry environments. One instance is that of gender differences at the Grammy Awards. Overall, from 2013 to 2021 the total number of Grammy male nominees was 86.6%, while that of female nominees was 13.4%. Furthermore, focusing on different award categories the percentage of male nominees is always greater than that of female nominees except for *best new artist*, where shares are more balanced-$-$54.55% male nominations compared to 45.45% female nominations (Smith et al. 2020).

**Fig. 4:**
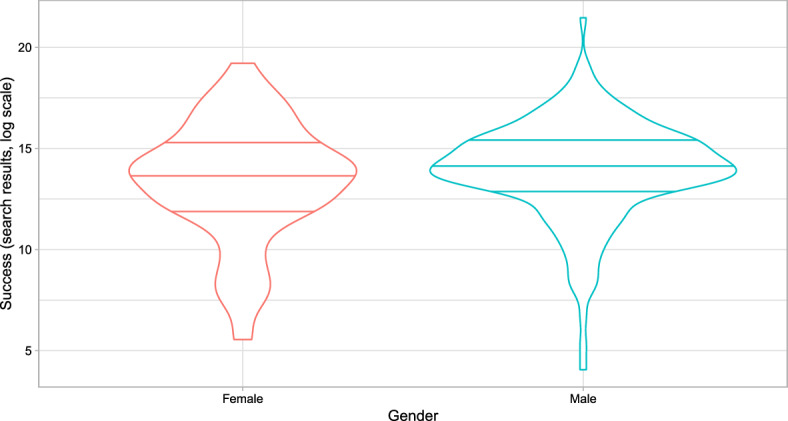
Distribution of popularity across gender. Both plots include reference lines for the first quartile (bottom), the median (center), and the third quartile (top)

Alternatively, this underrepresentation could imply that (on average) better known female artists are included in lineups bearing a greater impact on audiences. To address this biased effect, one should control for some metric of the popularity of performers. Looking at the number of results after running a Google search provides some interesting insights. Figure 4 shows the distribution of success (in a log scale) for female and male performers. Overall, it appears that the range of popularity for male performers is greater than that for female ones. We also see that success quantiles (as reference lines in the plots) indicate that male performers are more popular across the whole distribution. On the other hand, popularity is less concentrated for females than for males.

**Fig. 5:**
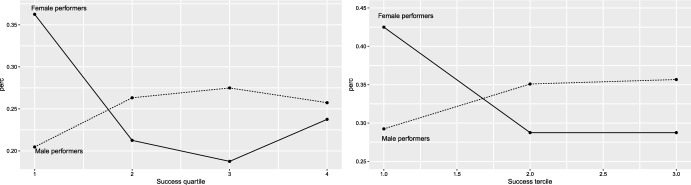
Percentage of performers in each quartile (left) and tercile (right) across gender

We further investigate this by looking at the percentage of female/male performers across the distribution of popularity. Figure 5 shows female(male) performers in each quartile(tercile) of the popularity distribution as a share of the total number of female(male) performers. It complements Fig. 4 in that it shows the greater concentration in the top quartile(tercile) for female artists. Now, it becomes apparent that the share of top popular female artists is greater than that of male artists.

Note that we have not find strong evidence supporting a greater effect for more popular artists. If this gender bias is related to success, it should enter the model as an interaction of popularity and gender (i.e., being female). However, note this would reduce the sample for this specific group making the estimates rather noisy, which advises against it.

Finally, a third potential explanation could be related to the nature of the streamed materials. The literature has acknowledged differences in gender in relation to performers, specifically in music videos (Conrad et al. 2009; Seidman 1992) as they often reproduce gender stereotypes—which tend to be genre-related (see Aubrey and Frisby 2011; Weitzer and Kubrin 2009). Specifically, female artists are more sexually objectified compared to male artists (Wallis 2011). The literature seems to agree that the content offered in music videos is different depending on the artist’s gender. Thus, it could be conjectured that the streaming of music videos is also biased by these differences.

### Limitations

Finally, with regard to the limitations of this study, mostly related to the empirical strategy, we would like to mention three. First, and with regard to the sample—i.e., performers playing at two established music festivals—we must acknowledge that results might not be unambiguously generalized. The type of cross-market effects we have measured need some scale and the creation of buzz, exposure and contagion through media (traditional and otherwise). This, in turn, limits the validity of the results to those contexts similar to the one analyzed in this paper. However, in this respect one must note there is a significant variability in the sample of performers analyzed (small acts, middle class performers and big stars) that becomes apparent when looking at the diverse lineups of the editions of the festivals considered.

Second, data on the outcome (i.e., the search index) are obtained as an unbiased random sample of video searches. This means the actual values we obtain change every time a retrieval request is made. These changes need not be important if artists generate enough Internet activity. However, one should acknowledge that the outcome variable is subject to sampling error that could affect the estimates most likely by driving them down.

Third, related to the previous point, by choosing weekly data we minimized the problem of zeroes for performers who generate low search activity. However, it creates an inherent ambiguity in the choice of the running variable (week to event) as the events always span over two values of the running variable. We tried to overcome this by using different definitions of the running variable, which provided robust results.

## Footnotes

1 *Euromonitor International* reports a monthly user base of over 20 million in Spain in 2020. Retrieved June 13, 2022 from https://www.euromonitor.com.

2 See https://www.apmusicales.com/ranking-festivales-2018/. Accessed on July 5th, 2022.

3 See website https://trends.google.es.

4 Available at https://github.com/GeneralMills/pytrends.

5 For instance, a query for the term *suede* returns, among others, the suggestions “Suede: Topic”, “Suede: Shoe store in Rome, Italy” and “Suede: Band”. We are interested in the latter.

6 Determined by mimicking variance evenly-spaced method using spacing estimators.

7 Estimates remain qualitatively unchanged when restricting the window before the live performance to 8 weeks.

8 These are robust to the choice of bandwidths. See Calonico et al. (2014).

9 See the Keychange manifesto at https://www.keychange.eu/.

10 It should be noted that any measure of success has a time-dependent component: if commercial success evolves over time, then any attempt to capture popularity should incorporate this fact. However, this (note that as performances take place over different years) along with the multidimensional nature of success complicates the task of finding a comprehensive metric. We consider our solution provides a reasonable approximation to success.

11 Searches were conducted on January 26 2023.
